# Acupoint Injection for Nonspecific Chronic Low Back Pain: A Systematic Review and Meta-Analysis of Randomized Controlled Studies

**DOI:** 10.1155/2020/3976068

**Published:** 2020-10-28

**Authors:** Guanli Xie, Tao Wang, Xiaoxia Tang, Xiaobo Guo, Yanfei Xu, Li Deng, Haomin Sun, Zhenxian Ma, Yuanliang Ai, Bo Jiang, Lvyu Li, Wen Luo, Wenze Huang, Yubo Xia, Heng Zhao, Xin Wang, Ying Guo, Jianglong Liao

**Affiliations:** ^1^Yunnan University of Chinese Medicine, Kunming, Yunnan, China; ^2^Kunming Municipal Hospital of Traditional Chinese Medicine, Kunming, Yunnan, China; ^3^Kunming Third People's Hospital, Kunming, Yunnan, China

## Abstract

**Introduction:**

Nonspecific chronic low back pain (NCLBP) became a public health and economic problem. Acupoint injection was used widely for patients with NCLBP. However, there were inconsistent results on the efficacy for these people. Therefore, this review was performed to systematically assess the efficacy and safety of acupoint injection.

**Materials and Methods:**

The literature sources were collected via EMBASE, Medline, CENTRAL, CINAHL, CNKI, VIP, Wanfang, and Sino-Med Database from their inception to October 13, 2019. Endnote X7, widely used document management software, was used to manage and screen the literature sources. Each record was screened according to the predetermined inclusion criteria by two review authors independently. Quality assessment tool, “Risk of table,” was used to assess the quality of the included studies according to the recommendation of the *Cochrane Handbook for Systematic Reviews of Interventions*. Data extraction was performed by one reviewer and verified by another reviewer. Any disagreement was addressed via consulting with a third reviewer in the abovementioned processes. All procedures were performed according to the *Cochrane Handbook for Systematic Reviews of Interventions*.

**Results:**

This review included 13 studies involving 1381 patients with NCLBP. Quantitative analysis results indicated that there is no sufficient evidence that acupoint injection can improve the pain of patients with low back pain based on two trails: Visual Analogue Scale (VAS: MD = −1.33, 95% confidence interval (95% CI) −3.30 to 0.64, *P*=0.18, random-effect model). When assessing the effectiveness of acupoint injection therapy, the results indicated that acupoint injection can improve the effective rate for nonspecific chronic low back pain (OR = 3.64, 95% CI 2.4 to 5.21, *P* < 0.0001, fixed-effect model).

**Conclusion:**

There is insufficient evidence to indicate that acupoint injection therapy could improve the pain for patients with NCLBP. However, the level of evidence was downgraded to “very low quality” because of the poor methodological quality and clinical heterogeneity. The results should be interpreted with caution. Higher quality RCTs with more appropriate comparison, more objective outcome instruments, and adequate follow-up periods are necessary to assess the efficacy of acupoint injection for NCLBP. The PROSPERO Research registration identifying number is CRD42019119158.

## 1. Introduction

The low back pain (LBP) refers to pain occurring between the 12th rib and the inferior gluteal fold [1, 2]. Epidemiologic studies reported that the prevalence of LBP is 23% to 84% [3]. Most patients (about 62%) still have pain after one year of the first episode of LBP, with 11-12% of those being disabled [3]. It was reported that 16% of CLBP patients were absent from work due to disability [4]. It is estimated that the direct medical cost of evaluating and treating LBP in the United States exceeds $33 billion annually. If the indirect costs of lost work are taken into account, it is more than $100 billion. It is a cause of Years lived with disability (YLDs) reported by the Global Burden of Disease Study (GBD) [5]. LBP is not only a major individual health problem but also became a public health and economic problem worldwide [6].

Chronic low back pain (CLBP) is defined as LBP over 3 months. The main symptom of CLBP is pain, and only 42% of patients were pain free after one year [7]. The causes of CLBP include biophysical factors, comorbidities, and poor mental health [8]. However, no cause can be found in most cases with low back pain. That is, the LBP is “nonspecific” [8–10].

Many interventions were used to improve the pain of CLBP, including surgery, drugs, and exercise [11]. However, there is no intervention that is better than others [11, 12]. Therefore, it is necessary to explore an effective and safe intervention for them.

Acupuncture has been used to treat CLBP for thousands of years [13, 14] and has been recommended by the American College of Physicians [15]. Based on the traditional acupuncture and injection therapy, acupoint injection was developed. It was a therapy that combined acupuncture and injection [16]. It was used to relieve pain for patients with CLBP widely in China and Korea [17]. Due to the usage of special forms and drugs, acupoint injection was also known as aqua acupuncture, herbal acupuncture, pharmacoacupuncture, water acupuncture, hydroacupuncture, or squirt cut. This therapy has the dual effects of acupuncture and medication. Specific substances injected into acupuncture points include vitamin [18–22], anesthetics [19, 20, 23, 24], glucocorticoid [19–21], Chinese herbal extracts [23–25], or other material [26, 27]. As with traditional acupuncture, acupoint injection stimulates acupoints to play a therapeutic role for CLBP [28]. Different from traditional acupuncture, acupoint injection also has therapeutic effects through substances, which was beneficial for patients with CLBP [17]. These substances work as a neuroprotectant, analgetic, or antioxidant. Acupoint injection enhanced stimulation on acupoints [17, 29]. It was recommended as an effective intervention that is also a simple, convenient, and time-saving treatment [30]. However, there are inconsistent results for the evidence for the effect of acupoint injection for patients with NCLBP [19, 21–24, 26]. Therefore, this systematic review was designed to assess the efficacy and safety of acupoint injection for NCLBP.

## 2. Materials and Methods

### 2.1. Design and Registration

The Research registration number of this study is CRD42019119158 (https://www.crd.york.ac.uk/PROSPERO/), and the protocol of this study was published previously [31]. For the literature research of this study, ethical authorization is not required.

### 2.2. Literature Sources

We searched eight online databases from their inception until October 13, 2019, with no language restrictions, including four English databases and four Chinese databases. The four English databases are Medline (PubMed), EMBASE (embase.com), Cochrane Central Register of Controlled Trials (CENTRAL) of Cochrane Library, and CINAHL via EBSCOhost. The four Chinese databases are the China National Knowledge Infrastructure (CNKI) database, Wanfang Database, Chinese Science and Technology Periodical (VIP) Database, and Sino-Med Database. The English terms such as “acupuncture point injection,” “acupoint injection,” “pharmacoacupuncture,” “hydroacupuncture,” “pharmacoacupuncture,” “LBP,” “low back pain,” and “chronic low back pain” were used individually or combined in English databases. The terms including “shu xue zhu she (acupoint injection),” “shui zhen (acupoint injection),” and “xue wei zhu she (acupoint injection)” were used in Chinese databases. The search strategy for Medline via PubMed is shown in Table 1 or acquired from the protocol of this study [31]. The full search strategy used in this study was submitted as supplementary material file 1.

### 2.3. The Inclusion Criteria and Exclusion Criteria of This Review

The studies were screened according to the inclusion criteria as follows: (1) patients: ≥18 years of age and suffering from nonspecific CLBP (persists for longer than 12 weeks); (2) intervention: acupuncture point injection therapy without limitation in drug and acupuncture point; (3) comparison: placebo-controlled or other nonacupoint injection therapy; (4) outcomes: the outcomes reported were not limited. The pain intensity was considered as the primary outcome such as the visual analogue scale (VAS) and proportion of pain-free patients; and (5) publication type: randomized controlled trials (RCTs). Exclusion was defined as follows: (1) patients: without nonspecific chronic low back pain; (2) intervention: nonacupoint injection was used, or acupoint injection was one part of a treatment package; (3) comparison: just different acupoint injection intervention methods, or treatment package in which the acupoint injection was used as one part of package acupoint injection; and (4) publication type: quasi-RCTs, non-RCTs such as case reports, systematic reviews, and letters to editors.

### 2.4. Data Acquisition and Processing

#### 2.4.1. Literature Screening

**Figure 1:**
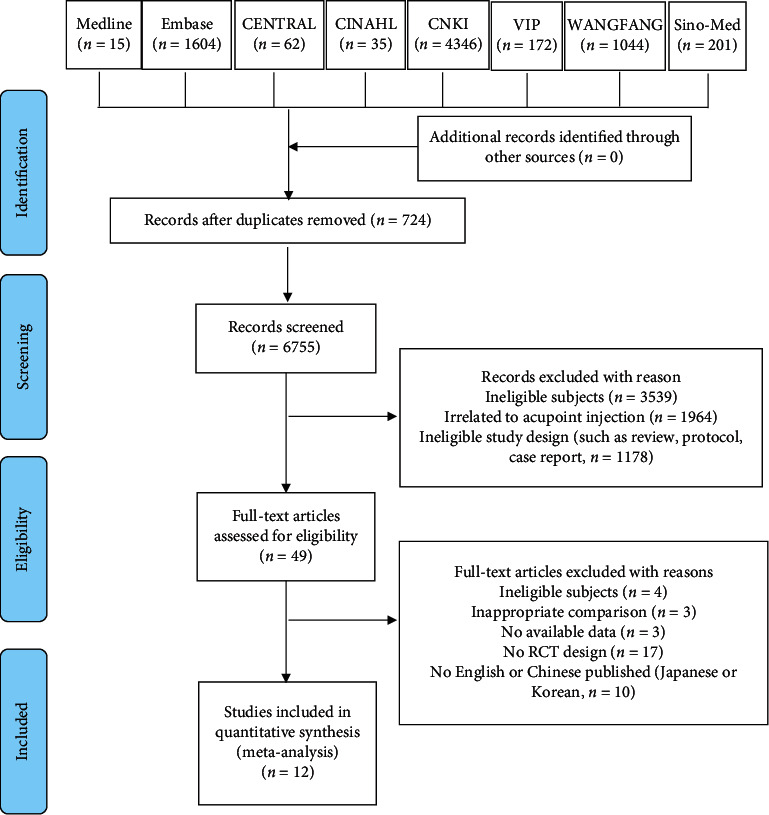
Flow diagram of the study selection process.

EndNote X7, widely used literature management software, was used to manage records. The duplicate records were removed according to the title, author, journal, and publication date. After that, two reviewer authors screened the records independently. The irrelevant literature was excluded according to the inclusion criteria and exclusion criteria via browsing the title and abstract of the article. Two independent review authors evaluate whether the study can be included according to predetermined selection criteria via reading the full text. In order to extract data accuracy and reliability, only full-text literature in English or Chinese were included in this study. The original author was contacted to obtain additional information when it is needed to make a judgment. Any inconsistencies were resolved with a third reviewer via discussion and consultation. The details of literature screening have been reported using the PRISMA flow diagram (Figure 1) according to the PRISMA checklist.

#### 2.4.2. Data Extraction and Management

A predesigned, verified, and formatted table was used to extract information. The extracted information includes five parts for each study. The information of the title, author, journal, and publishing year was extracted as basic information. The characteristics of participants of each study, including gender, age, inclusion, and exclusion criteria, were collected as the second part. The details of intervention used in the experimental and control groups such as the frequency, intervention time, follow-up period, injection content, dosage, and volume of injection at each point were extracted as the third part. The design information of each study, including the random sequence generation, randomization method, allocation concealment, blinding of participants and personnel, and blinding of outcome assessment was also extracted. The outcomes reported in each study such as outcome indicators, assessment tools, adverse events, and dropouts were extracted as the fifth part. The extraction of information was performed by two reviewers independently. One of them extracted the data and another checked the data. For the missing data, we have contacted the original author. For the studies that had more than two intervention groups, the data of intervention groups related to this review were extracted. For the studies that have two or more experimental groups, we combined the groups to create a single pairwise comparison.

#### 2.4.3. Assessment of Methodological Quality of Included Studies

The Cochrane Collaboration's tool, “Risk of bias,” was used to assess the quality of included studies by two reviewers independently [32]. Review authors' judgments involve answering a specific question for each entry. There are seven questions for reviewers to make a judgment. They are as follows: (1) Was the allocation sequence adequately generated? (2) Was the allocation adequately concealed? (3) Was knowledge of the allocated interventions adequately prevented during the study? (4) Were incomplete outcome data adequately addressed? (5) Are reports of the study of the suggestion of selective outcome reporting? (6) Was the study apparently of other problems that could put it at risk of bias? For each trial, the abovementioned questions were answered using “No,” “Unclear,” and “Yes.” For each question of each study, an answer “Yes” indicates a low risk of bias in this domain and an answer “No” indicates a high risk of bias in this domain. If there were very few details available to make a judgment of “low” or “high” risk, a judgment of “unclear” was made. Any inconsistencies were resolved with a third reviewer by discussion and consultation.

#### 2.4.4. Processing of Missing Data

For the missing data that were not reported in the manuscript, the original author was contacted to request additional data via e-mail. If the acquisition fails, only the available information and data were analyzed.

### 2.5. Data Analysis

Data analysis was performed by the Cochrane Review Manager (RevMan 5.3). The results were shown using mean difference (MD) with 95% confidence intervals (95% CIs) for continuous data. The results were shown using Odds Ratio (OR) with 95% CI for the enumeration data. The pooled effect was estimated using the fixed-effect model when the results indicate there is no significant heterogeneity between the included studies. If not, the pooled effect was estimated using the random-effect model. The chi-square test (*P* < 0.1) was used to assess the statistical heterogeneity, and I^2^ statistic was used to be quantified. If the I^2^ value is greater than 75%, the significant heterogeneity between the included studies was demonstrated according to the Cochrane Handbook for Systematic Reviews of Interventions [32]. The overall effect was synthesized using meta-analysis [33].

#### 2.5.1. Assessment of Publication Bias

The reporting bias was assessed using funnel plots for the outcomes, which were reported by more than ten studies [33].

#### 2.5.2. Grading the Quality of Evidence

The quality of evidence for outcomes was evaluated using the Grading of Recommendations Assessment, Development, and Evaluation (GRADE) system, which rates the quality as very low, low, moderate, or high levels [34].

## 3. Results

### 3.1. Literature Screening

A total of 7,479 records were obtained from online databases in this study. Among them, 724 records were deleted because they were identified as duplicate records. Other 6755 records were excluded after screening the abstracts and titles by two reviewers. Finally, twelve full texts were included for this review according to our inclusion/exclusion criteria. The details of the literature screening are described in Figure 1.

### 3.2. Study Characteristics

Table 2 shows the characteristics of the included studies. All twelve studies were designed as single-center studies. Nine studies were the pairwise comparison of intervention groups' design [18, 20–22, 25–27, 35]. The other three trials were multicomponent group intervention (three groups) design [23, 24, 36]. Five trials were followed up after intervention [18, 20, 26, 27, 35]. Also, the follow-up ranged from 12 weeks to 1 year. A total of 1381 participants with nonspecific chronic low back pain were included in this review. The number of participants in each trial ranged from 30 to 208. One of the twelve studies was from South Korea, while the others were from China. Only two trials reported the sources of patients [27, 36]. All included trials reported the subject selection criteria.

The points of acupoint used in each trial were diverse. The number of selected acupoints varies from 3 to 9. The Ashi acupuncture point in the low back was selected as the site of operation in nine of the twelve studies [19–24, 26, 35, 36]. Ten trials choose the points of the Taiyang Bladder Meridian of Foot (back Shu points) and five trials choose the points of the Governor Meridian (DU30). The BL23 was selected in seven trials. The BL25 was selected in seven trials. The BL40 was also selected in six trials [19–21, 23, 25, 26]. The GV03 was selected in five trials. Other points were selected including BL24, BL26, BL28, BL32, BL36, BL54, GB30, GB34, GB37, EX-B12, EX-B05, and SP9.

The substance used for acupoint injection was diverse from each other. A single substance or a mixture of substances was used in the studies. Three trials used single substance including Angelica injection [24, 36], Danshen injection [23], and Lidocaine injection [23]. The constituent substances of the mixture included Angelica injection [18, 22], Lidocaine injection, Oxygen-ozone [24, 36], Tong xi tong [22], Vitamin B1 [22], Vitamin B12 [18–22], Triamcinolone Acetonide Acetate [19–21], and *Cervus* and Cucumis Polypeptide injection [26]. Each acupoint was injected with 0.1 ml to 2 ml injectant. Each experimental group of included studies was treated once a day for four days [22], once a day for ten times [24, 26, 36], five times a week for 2 weeks [35], once a week for 2–4 weeks [20, 21], the next day at a time for ten days [25], the next day at a time for twenty days [18], twice a week for 4 weeks [27], and every three days for 20 days [23]. The intervention of the control group in each trials included electroacupuncture [19–21, 24, 36], acupuncture [23, 26, 35], oral administration of traditional Chinese medicine decoction [22], and placebo control [27].

The visual analogue scale (VAS) was used to assess the effect of acupoint injection for nonspecific chronic low back pain in three trials [18, 25, 27]. The MOS item short form health survey (SF-36), the Oswestry Disability Questionnaire (ODQ), and the straight leg raising test (SLRT) were used to assess the effect of acupoint injection for nonspecific chronic low back pain in one trial [27]. The subjective cure rate was used to assess the effect of acupoint injection in eleven trials [18–26, 35, 36].

### 3.3. Methodological Quality of Study

**Figure 2:**
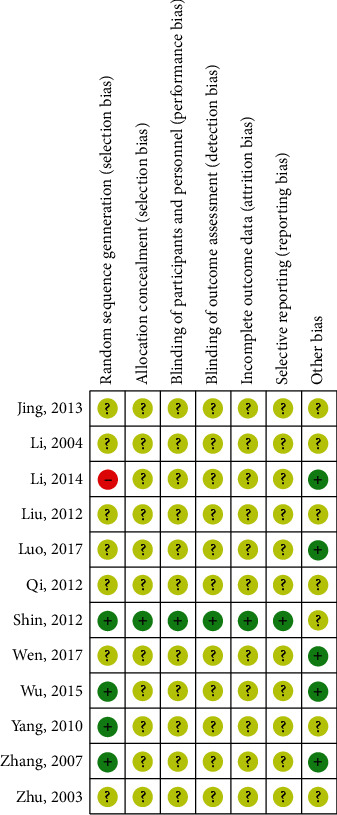
Risk of bias summary: review authors' judgments about each risk of bias item for each included study.

The risk of bias summary is shown in Figure 2 for the included studies. All trials reported random allocation implemented during the study. The random sequence generation methods reported included a random number generator in three studies [18, 24, 36] and Minitab software [27], for which the risk of bias of random number generation were “low risk.” One trial reported that the random number was generated according to the intervention in which the risk of bias of random number generation was “high risk.” Other seven trials did not describe the methods of random generation in which the risk of bias was unclear. One study reported allocation concealment [27]. The risk of performance bias, the risk of detection bias, the risk of attrition bias, and the risk of reporting bias were judged as “unclear risk” of eleven trials because there is insufficient information about these domains to permit the judgment of “Yes” or “No.” One study was judged as “low risk” in terms of performance bias, detection bias, attrition bias, and reporting bias [27]. Five trials were judged as “low risk” in terms of other bias because a comparison of baseline data before treatment was reported [18–20, 25, 36], while the other seven trials were judged as “unclear risk” because there is no enough information to permit the judgment of “Yes” or “No” [21–24, 26, 27, 35].

### 3.4. The Effectiveness of Acupoint Injection in Terms of Pain for Patients with NCLBP

**Figure 3:**
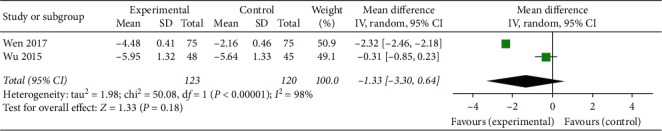
Meta-analysis of acupoint injection therapy on pain.

Three of twelve trials reported the effects of acupoint injection in terms of pain for patients with NCLBP using the VAS [18, 25, 27], while we only obtain the original data of the VAS from two studies [18, 25]. Thus, only two studies were included in the meta-analysis for the primary outcomes [18, 25]. The results demonstrated that there is not enough evidence to indicate that acupoint injection can improve the pain of patients with nonspecific CLBP using the VAS (*n* = 243, MD = −1.33, 95% CI −3.30 to 0.64, *P*=0.18, the random-effect model), with a substantial heterogeneity (*I*^2^ = 98%) as shown in Figure 3.

The effectiveness of acupoint injection in terms of subjective effective rate for patients with NCLBP.

**Figure 4:**
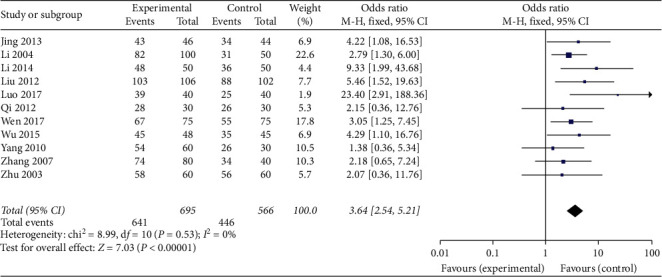
Meta-analysis of acupoint injection therapy on the subjective effective rate.

Eleven trials reported the effect of acupoint injection therapy using a subjective effective rate for NCLBP [18–26, 35, 36]. The results demonstrated that acupoint injection can improve the symptoms of low back pain using a subjective effective rate (*n* = 1261, OR = 3.64, 95% CI 2.54 to 5.21, *P* < 0.00001, the fixed-effect model), with homogeneity (*I*^2^ = 0%) as shown in Figure 4.

### 3.5. Dropout

The number and reasons for dropout were reported in one study [27]. The intension-to-treat (ITT) analysis and per-protocol (PP) analysis were carried out in one trial [27]. There was no information of dropout reported in the other eleven studies [18–26, 35, 36].

### 3.6. Adverse Effects

The adverse reactions were reported in two trials which reported that skin flair, edema, and skin rash have occurred in the experimental group and skin rash, headache, hand-foot tingling occurred in the placebo control group [23, 27]. The original author also reported that “all adverse reactions disappeared after the study without the use of antihistamines or medical interventions” [27]. Other studies did not report there were adverse events.

### 3.7. Publication Bias

The publication bias was performed for the subjective effective rate reported by eleven trials in this review [37]. Although the funnel plot is symmetrical (supplementary material file 2), we also considered that there is reporting bias due to language bias and poor methodological quality.

### 3.8. Grading the Quality of Evidence

The quality of evidence for outcomes was evaluated using the Grading of Recommendations Assessment, Development, and Evaluation (GRADE) system, which rates the quality as very low, low, moderate, or high levels [34]. For this review, the quality of evidence was considered as very low because of the low methodology quality of the included studies.

## 4. Discussion

This study assessed the efficacy of acupoint injection therapy on pain for patients with NCLBP collating evidence from RCTs. A total of 12 RCTs were included in this review involving 1381 patients. Based on our meta-analysis, there was not enough evidence to indicate that acupoint injection can improve pain for patients with nonspecific CLBP. However, acupuncture point injection therapy can improve the subjective effective rate for patients with nonspecific CLBP. The adverse reactions include skin flair, edema, and skin rash. All adverse reactions disappeared after the study without the use of antihistamines or medical interventions.

There was no reason to upgrade the strength of evidence. However, due to the limitations in the design and implementation inherent imprecision (low quality of included studies) and publication bias (there were less than ten studies reported the primary outcome), the level of evidence was downgraded. Finally, the quality of the evidence was downgraded to “very low quality” for the primary outcomes of pain.

### 4.1. Strengths and Limitations

This review and meta-analysis was performed according to the Cochrane Handbook for Systematic Reviews of Intervention. The formulation of the search strategy for each online database is helpful for the comprehensiveness and extensiveness of literature collection. The study protocol predetermined was helpful to select studies, extract data, and synthesize data. According to the study protocol, two independent review authors complete the literature screening, data extraction, quality assessment of the included trials. When encountering a contradiction, we discuss with a third reviewer to make a final decision. These methods reduced bias and transcription errors.

At the same time, there are some limitations we should note. The different interventions, different acupuncture points, intervention frequency, and duration lead to the high clinical heterogeneity between included studies. Another source of clinical heterogeneity is the diversification of the substance injected into points. The Angelica injection, Lidocaine injection, Oxygen-ozone, Tong xi tong, Vitamin B_1_, Vitamin B_12_, Triamcinolone Acetonide Acetate, and *Cervus* and Cucumis Polypeptide injection were used in different trials. The type of acupuncture point injection was different between trials including single acupoint injection and combined with acupuncture, electroacupuncture, or functional training. All these factors make it difficult to confirm a distinct relationship between acupuncture point injection and the improvement of pain. Although eleven of the included twelve trials reported the effectiveness of acupoint injection for the patients with NCLBP, the subjective instrument used in trials challenged the reliability and validity of the results. Only full-text records in English or Chinese were included, and unpublished materials were not retrieved in this study, which will also bring limitations to the results.

### 4.2. Clinical Relevance and Future Directions

There is no sufficient evidence to indicate that acupuncture point injection can improve the pain of nonspecific CLBP. Acupuncture can improve the subjective effective rate for patients with nonspecific CLBP. But, the results have been challenged due to the subjective instruments used in the trials.

In future studies, the following issues need to be noted. To explore the optimal intervention intensity, frequency, and dosage of substances for acupoint injection may be the future research direction. The intervention of the control group should be set according to the purpose of research. For the studies whose aim is to demonstrate the effect of the injected drugs, the control groups should be placebo control. For the studies whose aim is to demonstrate the effect of the acupuncture points, the control groups should be nonacupuncture point control. The selection of instruments to assess the clinical effect of acupoint injection therapy is important. Assessment tools that are more objective and sensitive should be used in future studies. The long-term outcomes should be of concern, and other outcomes such as quality of life and recurrence of low back pain also should be of concern. The factors that influence the effects such as profession and daily activities, should be considered in future trials. Finally, and most importantly, the CONSORT guidelines should be followed to report the trials to allow a better evaluation of the methodological quality. Higher quality RCTs with more appropriate comparison, more objective outcome instruments, and adequate follow-up periods are necessary to assess the efficacy of acupoint injection for NCLBP.

## 5. Conclusions

In conclusion, there is no sufficient evidence to indicate that acupuncture point injection can improve pain for patients with nonspecific CLBP using the VAS. The acupuncture point injection may have a positive effect for the subjective effective rate. The adverse events reported included skin flair, edema, and skin rash. These adverse events disappeared spontaneously without intervention. However, due to the differences in the acupoints, drugs, injection doses, frequencies, durations of acupuncture point injection, and the poor quality of the included studies, caution is needed when interpreting these results. Higher quality RCTs with more appropriate comparison, more objective outcome instruments, and adequate follow-up periods are necessary to assess the efficacy of acupoint injection for NCLBP.

## Figures and Tables

**Table 1:** Search strategy for Medline via PubMed.

No.	Search items
#1	“dorsalgia” [tw]
#2	“Back Pain”[Mesh]
#3	(“backache” or “back pain”) [tw]
#4	(“lumbar adj pain”) [tw]
#5	“coccyx”[tw]
#6	“coccydynia”[tw]
#7	“sciatica”[tw]
#8	“sciatic neuropathy” [Mesh]
#9	“spondylosis”[tw]
#10	“lumbago”[tw]
#11	“back disorder$”[tw]
#12	“Back Muscles”[Mesh]
#13	#1 OR #2 OR #3 OR #4 OR #5 OR #6 OR #7 OR #8 OR #9 OR #10 OR #11 OR #12
#14	“Acupuncture Therapy”[Mesh] or “Acupuncture, Ear”[Mesh] or “Acupuncture Points”[Mesh] or “Acupuncture Analgesia”[Mesh] or “Acupuncture”[Mesh]
#15	“Acupressure”[Mesh]
#16	“Electroacupuncture”[Mesh]
#17	“Meridians”[Mesh]
#18	“Moxibustion”[Mesh]
#19	“Acupuncture”[tw]
#20	“acupressure$”[tw]
#21	(“electroacupuncture” or “electro acupuncture” or “electro-acupuncture”) [tw]
#22	“meridian$”[tw]
#23	“mox$”[tw]
#24	“needling”[tw]
#25	(“acu-point$” or “acupoint$”)[tw]
#26	“acupoint$”[tw]
#27	“shu”[tw]
#28	(“shiatsu” or “tui na”) [tw]
#29	#14 OR #15 OR #16 OR #17 OR #18 OR #19 OR #20 OR #21 OR #22 OR #23 OR #24 OR #25 OR #26 OR #27 OR #28
#30	“injection” [tw]
#31	“injections” [tw]
#32	#30 OR #31
#33	#29 AND #32
#34	randomized controlled trial [pt]
#35	controlled clinical trial [pt]
#36	randomized [tiab]
#37	placebo [tiab]
#38	clinical trials as topic [mesh: noexp]
#39	randomly [tiab]
#40	trial [ti]
#41	#34 OR #35 OR #36 OR #37 OR #38 OR #39 OR #40
#42	animals [mh] NOT humans [mh]
#43	#41NOT #42
#44	#13 AND #33 AND #43

**Table 2:** Characteristics of included studies in this systematic review.

Author, year	Age range	Participants (M/F)	Interventions	Frequency and duration of the acupoint injection	Acupoint injection drug, dose	Acupoints injected	Outcomes/measure(s)
Jing, 2013 [26]	18–75	90 (43/47)	EG: Acupoint injection + TDP CG: Acupuncture	EG: 15 min-2 min/day, once a day or alternate dose, 10 dayS CG: 40 min/day, once a day, 10 dayS	*Cervus* and Cucumis Polypeptide injection 4 ml, 0.5–1 ml/point	BL32, BL36, BL40, BL54, BL57, GB37, GB30, SP9, Ashi point	Subjective cure rate

Li et al., 2004 [23]	25–75	150 (63/87)	EG1: Acupoint injection (2% lidocaine) EG2: Acupoint injection (Danshen injection) CG: Acupuncture	EG1: Every three days, 20 days EG2: Every three days, 20 days CG: every other day, 20 days	EG1: 2% lidocaine EG2: Danshen injection	BL23, BL24, BL25, BL40, GB30, GB34, EX-B05, Ashi point	Subjective cure rate

Li, 2014 [19]	22–76	100 (59/41)	EG: Acupoint injection + Electroacupuncture CG: Electroacupuncture	EG: Acupoint injection: Once/5–7 days Electroacupuncture: 25 min one time, once a day, 6 days as a session, 2–4 sessions CG: Electroacupuncture	2% lidocaine 4 ml + triamcinolone acetonide acetate 12.5 mg + vitamin B12 2 ml	BL23, BL25, BL40, BL54, GV03, Ashi point	Subjective cure rate

Liu et al., 2012 [35]	Did not report	208 (did not report)	EG: Acupoint injection + acupuncture CG: Acupuncture	EG: Acupuncture: 20 min/day, 5 times/week, 2 weeks; acupoint injection: Once a week, 2 weeks CG: 20 min/day, 5 times/week, 2 weeks	Diprospan 5 mg + mecobalamin 0.5 mg + 0.9% NaCl 10 ml	BL23, BL28, Ashi point	Subjective cure rate

Luo and Han, 2017 [20]	23–78	80 (65/15)	EG: Acupoint injection + Electroacupuncture. CG: Electroacupuncture	EG: Acupuncture: 25 min/day, 6 times/week, 2–4 weeks; acupoint injection: Once a week, 2–4 weeks CG: 25 min/day, 6 times/week, 2–4 weeks	2% lidocaine 4 ml + triamcinolone acetonide acetate 12.5 mg + vitamin B_12_ + 2 ml	BL23, BL25, BL40, BL54, GV03, Ashi point	Subjective cure rate; Patient satisfaction

Qi, 2012 [21]	22–78	60 (39/21)	EG: Acupoint injection + Electroacupuncture CG: Electroacupuncture	EG: Acupuncture: 25 min/day, 6 times/week, 2–4 weeks; acupoint injection: once a week, 2–4 weeks CG: 25 min/day, 6 times/week, 2–4 weeks	2% lidocaine 4 ml + triamcinolone acetonide acetate 12.5 mg + vitamin B1 2.2 ml	BL23, BL25, BL40, BL54, GV03, Ashi point	Subjective cure rate

Shin et al., 2012 [27]	18–65	60 (27/33)	EG: Acupoint injection CG: sham injection	EG: Twice a week for 4 weeks for a total of 8 treatment sessions CG: twice a week for 4 weeks for a total of 8 treatment sessions	EG: In the BVA group, the bee venom was diluted 1 : 2000, and a volume of 0.1 ml was injected into each acupoint. The total injected volume per participant was set at 0.6 ml (0.1 ml × 6 points) CG: Equal amounts of normal saline were injected into each acupoint (0.1 ml × 6 points)	BL23, BL24, BL25	VAS, SF-36, ODQ, SLRT

Wen et al., 2017 [25]	40–75	150 (85/65)	EG: Acupoint injection + function exercise CG: function exercise + voltaren (oral)	EG: Function exercise: did not report; Acupoint injection: once two days CG: function exercise: did not report; Voltaren (oral): Two pills each time, once a day	Danhong injection 2 ml each point	GV03, BL23, BL40, BL32	VAS; subjective cure rate

Wu et al., 2015 [18]	24–56	93 (52/41)	EG: Acupoint injection + traditional Chinese medicine bone setting technique CG: Lumbar traction	EG: Acupoint injection: once a day, 20 times as a session; Traditional Chinese medicine bone setting technique: Once every two days, 20 times as a session CG: 15–40 minutes/day	0.5 mg vitamin B_12_ (1 ml) + compound Angelica injection (2 ml) + 0.9% NaCl (2 ml)	Back Shu point	Subjective cure rate; VAS

Yang and Wang, 2010 [24]	Did not report	90 (did not report)	EG1: Acupoint injection (Angelica injection) EG2: Acupoint injection (O_3_ injection) CG: Electroacupuncture	EG1: Once a day, ten times as a session, one session EG2: Once a day, ten times as a session, one session CG: Once a day, ten times as a session, one session	EG1: Angelica injection (0.5–1 ml) EG2: 1% lidocaine + 30 *μ*g/ml oxygen-ozone 3–5 ml	BL24, BL25, BL26, Ashi point	Subjective cure rate

Zhang et al., 2007 [36]	Did not report	120 (65/55)	EG1: Acupoint injection (Angelica injection) EG2: Acupoint injection (O_3_ injection) CG: Electroacupuncture	EG1: Once a day, ten times as a session, one session EG2: Once a day, ten times as a sesson, one session CG: Once a day, ten times as a sesson, one session	EG1: Angelica injection 0.5–1 ml EG2: 30 *μ*g/ml oxygen-ozone 3–5 ml	BL24, BL25, BL26, Ashi point	Subjective cure rate

Zhu, 2003 [22]	38–72	180 (did not report)	EG1: Acupoint injection; EG2: Oral Chinese medicine; CG: The five elements of haci needle + anti-inflammatory agent	EG1: Once a day, four times as a sesson, one session EG2: Once a day, four times as a sesson, one session CG: 20 minutes/day, once a day, ten times as a session, one session	Tongxitong 40 mg + Angelica injection 4 ml + vitamin B_1_ 200 mg + vitamin B12 100 mg	GV03, EX-B02, GB34, Ashi point	Subjective cure rate

VAS, visual analogue scale.

## Data Availability

The datasets used and/or analyzed during the current study are available from the corresponding author on reasonable request.

## Abbreviations

LBP: Low back pain
CLBP: Chronic low back pain
YLDs: Years lived with disability
DALYs: Disability-adjusted life years
RCTs: Randomized controlled trials
VAS: Visual analogue scale
CNKI: The China National Knowledge Infrastructure
VIP: The Chinese Science and Technology Periodical
ROB: Risk of bias
VAS: Visual analogue scale.

## Supplementary Materials

Supplementary Materials Supplementary file 1: search Strategy for each database used in this review. Supplementary file 2: funnel plots for the subjective effective rate.
