# Supplementary material for: Acupoint Injection for Nonspecific Chronic Low Back Pain: A Systematic Review and Meta-Analysis of Randomized Controlled Studies
Source: Evid Based Complement Alternat Med. 2020 Oct 28;2020:3976068. doi: 10.1155/2020/3976068 (PMC7641697; doi:10.1155/2020/3976068)
Supplement: Supplementary Materials — Supplementary file 1: search Strategy for each database used in this review. Supplementary file 2: funnel plots for the subjective effective rate. [file 3976068.f1.zip › 3976068.f1/supplementary material file 1.Search Strategy for all databases.docx]

**Supplementary material file: Search Strategy for each database used in this review**

**1. Medline via PubMed**

#1 “dorsalgia” [tw]

#2 “Back Pain”[Mesh]

#3 (“backache” or “back pain”) [tw]

#4 (“lumbar adj pain”) [tw]

#5 “coccyx”[tw]

#6 “coccydynia”[tw]

#7 “sciatica”[tw]

#8 “sciatic neuropathy” [Mesh]

#9 “spondylosis”[tw]

#10 “lumbago”[tw]

#11 “back disorder$”[tw]

#12 “Back Muscles”[Mesh]

#13 #1 OR #2 OR #3 OR #4 OR #5 OR #6 OR #7 OR #8 OR #9 OR #10 OR #11 OR #12

#14 “Acupuncture Therapy”[Mesh] or “Acupuncture, Ear”[Mesh] or “Acupuncture Points”[Mesh] or “Acupuncture Analgesia”[Mesh] or “Acupuncture”[Mesh]

#15 “Acupressure”[Mesh]

#16 “Electroacupuncture”[Mesh]

#17 “Meridians”[Mesh]

#18 “Moxibustion”[Mesh]

#19 “Acupuncture”[tw]

#20 “acupressure$”[tw]

#21 (“electroacupuncture” or “electro acupuncture” or “electro-acupuncture”) [tw]

#22 “meridian$”[tw]

#23 “mox$”[tw]

#24 “needling”[tw]

#25 (“acu-point$” or “acupoint$”)[tw]

#26 “acupoint$”[tw]

#27 “shu”[tw]

#28 (“shiatsu” or “tui na”) [tw]

#29 #14 OR #15 OR #16 OR #17 OR #18 OR #19 OR #20 OR #21 OR #22 OR #23 OR #24 OR #25 OR #26 OR #27 OR #28

#30 “injection” [tw]

#31 “injections” [tw]

#32 #30 OR #31

#33 #29 AND #32

#34 randomized controlled trial [pt]

#35 controlled clinical trial [pt]

#36 randomized [tiab]

#37 placebo [tiab]

#38 clinical trials as topic [mesh: noexp]

#39 randomly [tiab]

#40 trial [ti]

#41 #34 OR #35 OR #36 OR #37 OR #38 OR #39 OR #40

#42 animals [mh] NOT humans [mh]

#43 #41NOT #42

#44 #13 AND #33 AND #43

**2.EMBASE via emmbase.com**

#1 ‘dorsalgia’:ti:ab

#2 ‘Back Pain’/exp

#3 (‘backache’ or ‘back pain’):ti:ab

#4 (‘lumbar adj pain’):ti:ab

#5 ‘coccyx’:ti:ab

#6 ‘coccydynia’:ti:ab

#7 ‘sciatica’:ti:ab

#8 ‘sciatic neuropathy’/exp

#9 ‘spondylosis’:ti:ab

#10 ‘lumbago’:ti:ab

#11 ‘back disorder$’:ti:ab

#12 ‘Back Muscles’/exp

#13 #1 OR #2 OR#3 OR #6 OR #9 OR #10 OR #11 OR #12

#14 ‘Acupuncture Therapy’/exp or ‘Acupuncture, Ear’/exp or ‘Acupuncture Points’/exp or ‘Acupuncture Analgesia’/exp or ‘Acupuncture’/exp

#15 ‘Acupressure’/exp

#16 ‘Electroacupuncture’/exp

#17 ‘Meridians’/exp

#18 ‘Moxibustion’/exp

#19 ‘Acupuncture’:ti:ab

#20 ‘acupressure$’:ti:ab

#21 (‘electroacupuncture’ or ‘electro acupuncture’ or ‘electro-acupuncture’):ti:ab

#22 ‘meridian$’:ti:ab

#23 ‘mox$’:ti:ab

#24 ‘needling’:ti:ab

#25 (‘acu-point’ or ‘acupoint’):ti:ab

#26 ‘acupoint$’:ti:ab

#27 ‘shu’:ti:ab

#28 (‘shiatsu’ or ‘tui na’):ti:ab

#29 #14 OR #15 OR #16 OR #17 OR #18 OR #19 OR #20 OR #21 OR #22 OR #23 OR #24 OR #25 OR #26 OR #27 OR #28

#30 ‘injection’:ti:ab

#31 ‘injections’:ti:ab

#32 #30 OR #31

#33 #29 AND #32

#34 ‘randomized controlled trial’:ti:ab

#35 ‘controlled clinical trial’:ti:ab

#36 ‘randomized’:ti:ab

#37 ‘placebo’:ti:ab

#38 ‘clinical trials as topic’/exp

#39 ‘randomly’:ti:ab

#40 ‘trial’:ti

#41 #34 OR #35 OR #36 OR #37 OR #38 OR #39 OR #40

#42 animals/exp NOT humans/exp

#43 #41 NOT #42

#44 #13 AND #33 AND #43

**3.CENTRAL**

#1 “dorsalgia”:ti,ab,kw

#2 [mh ^“Back Pain”]

#3 (“backache” or “back pain”):ti,ab,kw

#4 (“lumbar adj pain”):ti,ab,kw

#5 “coccyx”:ti,ab,kw

#6 “coccydynia”:ti,ab,kw

#7 “sciatica”:ti,ab,kw

#8 [mh ^“sciatic neuropathy”]

#9 “spondylosis”:ti,ab,kw

#10 “lumbago”:ti,ab,kw

#11 “back disorder*”:ti,ab,kw

#12 [mh “Back Muscles”]

#13 #1 OR #2 OR #3 OR #4 OR #5 OR #6 OR #7 OR #8 OR #9 OR #10 OR #11 OR #12

#14 [mh “Acupuncture Therapy”] or [mh “Acupuncture, Ear”] or [mh “Acupuncture Points”] or [mh “Acupuncture Analgesia”] or [mh “Acupuncture”]

#15 [mh “Acupressure”]

#16 [mh “Electroacupuncture”]

#17 [mh “Meridians”]

#18 [mh “Moxibustion”]

#19 “Acupuncture”:ti,ab,kw

#20 “acupressure*”:ti,ab,kw

#21 (“electroacupuncture” or “electro acupuncture” or “electro-acupuncture”):ti,ab,kw

#22 “meridian*”:ti,ab,kw

#23 “mox*”:ti,ab,kw

#24 “needling”:ti,ab,kw

#25 (“acu-point*” or “acupoint*”):ti,ab,kw

#26 “acupoint*”:ti,ab,kw

#27 “shu”:ti,ab,kw

#28 (“shiatsu” or “tui na”):ti,ab,kw

#29 #14 OR #15 OR #16 OR #17 OR #18 OR #19 OR #20 OR #21 OR #22 OR #23 OR #24 OR #25 OR #26 OR #27 OR #28

#30 “injection”:ti,ab,kw

#31 “injections”:ti,ab,kw

#32 #30 OR #31

#33 #29 AND #32

#34 randomized controlled trial:pt

#35 controlled clinical trial:pt

#36 randomized:ti,ab

#37 placebo:ti,ab

#38 [mh “clinical trials as topic”]

#39 randomly:ti,ab

#40 trial:ti

#41 #34 OR #35 OR #36 OR #37 OR #38 OR #39 OR #40

#42 [mh animals] NOT [mh humans]

#43 #41 NOT #42

#44 #13 AND #33 AND #43

**4. CINAHL via Ebsco**

S1 TI (dorsalgia ) or AB (dorsalgia)

S2 MH (“Back Pain+”)

S3 TI (backache or back pain) or AB (backache or back pain)

S4 TI (lumbar adj pain) or AB (lumbar adj pain)

S5 TI (coccyx) or AB (coccyx)

S6 TI (coccydynia) or AB (coccydynia)

S7 TI (sciatica) or AB (sciatica)

S8 MH (“sciatic neuropathy+”)

S9 TI (spondylosis) or AB (spondylosis)

S10 TI (lumbago) or AB (lumbago)

S11 TI (back disorder*) or AB (back disorder*)

S12 MH (“Back Muscles+”)

S13 S1 OR S2 OR S3 OR S4 OR S5 OR S6 OR S7 OR S8 OR S9 OR S10 OR S11 OR S12

S14 (MH “Acupuncture Therapy+”) or (MH “Acupuncture, Ear”) or (MH “Acupuncture Points+”)/exp or (MH “Acupuncture Analgesia”) or (MH “Acupuncture+”)

S15 (MH “Acupressure+”)

S16 (MH “Electroacupuncture+”)

S17 (MH “Meridians+”)

S18 (MH “Moxibustion+”)

S19 TI (Acupuncture) or AB (Acupuncture)

S20 TI (acupressure*) or AB (acupressure*)

S21 TI(electroacupuncture or electro acupuncture or electro-acupuncture) or AB (electroacupuncture or electro acupuncture or electro-acupuncture)

S22 TI (meridian*) or AB (meridian*)

S23 TI (mox*) or AB (mox*)

S24 TI (needling) or AB (needling)

S25 TI (acu-point* or acupoint*) or AB (acu-point* or acupoint*)

S26 TI (acupoint*) or AB (acupoint*)

S27 TI (shu) or AB (shu)

S28 TI (shiatsu or tui na) or AB (shiatsu or tui na)

S29 S14 OR S15 OR S16 OR S17 OR S18 OR S19 OR S20 OR S21 OR S22 OR S23 OR S24 OR S25 OR S26 OR S27 OR S28

S30 TI (injection) or AB (injection)

S31 TI (injections) or AB (injections)

S32 S30 OR S31

S33 S29 AND S32

S34 PT (randomized controlled trial)

S35 PT (controlled clinical trial)

S36 TI (randomized) or AB (randomized)

S37 TI (placebo) or AB (placebo)

S38 (MH “clinical trials as topic+”)

S39 TI (randomly) or AB (randomly)

S40 TI (trial)

S41 S34 OR S35 OR S36 OR S37 OR S38 OR S39 OR S40

S42 SU human

S43 S13 AND S33 AND S41 AND S42

**5. CNKI：**

(SU=‘水针’+‘穴位注射’+‘穴位疗法’+‘针灸’ OR TI=‘水针’+‘穴位注射’+‘穴位疗法’+‘针灸’ OR KY=‘水针’+‘穴位注射’+‘穴位疗法’+‘针灸’ OR AB=‘水针’+‘穴位注射’+‘穴位疗法’+‘针灸’) AND (SU=‘腰痛’+ ‘腰背痛’+‘腰椎间盘突出’+‘背痛’+‘腰腿痛’ OR TI=‘腰痛’+‘腰背痛’+‘腰椎间盘突出’+‘背痛’+‘腰腿痛’ OR KY=‘腰痛’+‘腰背痛’+‘腰椎间盘突出’+‘背痛’+‘腰腿痛’ OR AB=‘腰痛’+‘腰背痛’+‘腰椎间盘突出*’+‘背痛’+‘腰腿痛’)

**6. Sino-Med Database**

(( "水针"[摘要:智能] OR "穴位注射"[摘要:智能]) OR( "水针"[关键词:智能] OR "穴位注射"[关键词:智能]) OR( "水针"[加权:扩展] OR "穴位注射"[加权:扩展]) )AND( ( "腰痛"[摘要:智能] OR "腰背痛"[摘要:智能] OR "腰腿痛"[摘要:智能]) OR( "腰痛"[关键词:智能] OR "腰背痛"[关键词:智能] OR "腰腿痛"[关键词:智能]) OR( "腰痛"[加权:扩展] AND "腰背痛 OR 腰腿痛"[加权:扩展]))

**7.WanFang：**

( ((题名或关键词:(腰痛)+题名或关键词:(腰背痛)+题名或关键词:(腰腿痛)+主题:(腰痛)+主题:(腰背痛)*主题:(腰腿痛)))AND((题名或关键词:(水针)+题名或关键词:(穴位注射)+主题:(水针)+主题:(穴位注射))))*Date:-2019

**8.VIP：**

(M=水针+穴位注射) AND (M=腰椎间盘突出症+lumbar disc herniation+lumbar disc protrusion+lumbar disk herniation+lumbar herniated disk+lumbar intervertebral disc herniation+lumbar intervertebral disc prolapse+prolapse of lumbar intervertebral disc+腰背疼痛+腰椎盘突出症+腰椎盘突出+腰脱+椎间盘突出+腰腿痛+腰椎间突出症+下腰痛+下背痛+腰椎间盘纤维环破裂症+腰突症+椎间盘膨出+腰椎间盘膨出+腰椎椎间盘突出+下腰疼+腰椎间盘脱出症+腰痛+腰椎间盘脱出+腰背痛+腰椎问盘突出症+腰间盘突出症+椎间盘突出症+腰椎间盘突出+远红外热像图+腰间盘突出) AND (M=随机+对照+临床)
