# Supplementary figures and images for: Acupoint Injection for Nonspecific Chronic Low Back Pain: A Systematic Review and Meta-Analysis of Randomized Controlled Studies
Source: Evid Based Complement Alternat Med. 2020 Oct 28;2020:3976068. doi: 10.1155/2020/3976068 (PMC7641697; doi:10.1155/2020/3976068)

Supplementary materials file 2: funnel plots for the subjective effective rate


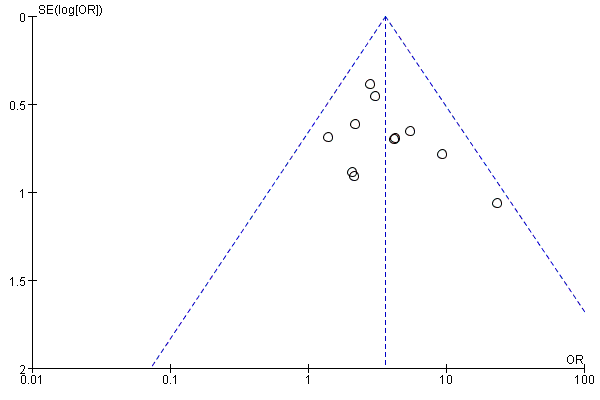

Supplement: Supplementary Materials — Supplementary file 1: search Strategy for each database used in this review. Supplementary file 2: funnel plots for the subjective effective rate. [file 3976068.f1.zip › 3976068.f1/supplementary file 2.docx]
